# A suicide attentional bias as implicit cognitive marker of suicide vulnerability in a high-risk sample

**DOI:** 10.3389/fpsyt.2024.1406675

**Published:** 2024-08-07

**Authors:** Juliane Brüdern, Lena Spangenberg, Maria Stein, Helena Gold, Thomas Forkmann, Katarina Stengler, Heide Glaesmer

**Affiliations:** ^1^ Department of Medical Psychology and Medical Sociology, University of Leipzig, Leipzig, Germany; ^2^ Department of Clinical Psychology and Psychotherapy, University of Bern, Bern, Switzerland; ^3^ Translational Research Center, University Hospital of Psychiatry and Psychotherapy, University of Bern, Bern, Switzerland; ^4^ Department of Clinical Psychology and Psychotherapy, University of Duisburg-Essen, Duisburg-Essen, Germany; ^5^ Department of Psychiatry, Psychotherapy and Psychosomatics, Helios Park Hospital Leipzig, Leipzig, Germany

**Keywords:** suicide attentional bias, suicide stroop task, suicide ideation, suicide attempt, behavioral test, implicit marker

## Abstract

**Introduction:**

Suicide risk assessment based on self-report questionnaires is considered as problematic because risk states are dynamic and at-risk individuals may conceal suicidal intentions for several reasons. Therefore, recent research efforts increasingly focus on implicit risk markers such as the suicide attentional bias (SAB) measured with the Suicide Stroop Task (SST). However, most SST studies failed to demonstrate a SAB in individuals with suicide risk and repeatedly demonstrated insufficient psychometrics of the SST. This study aimed to investigate a SAB using a modified SST (M-SST) and to test its psychometric properties.

**Method:**

We compared n = 61 healthy controls and a high-risk inpatient sample of n = 40 suicide ideators and n = 40 suicide attempters regarding interference scores of positive, negative and suicide-related words. Interference scores were calculated by subtracting the mean reaction time (mean RT) of the neutral words from the mean RT of the suicide-related words (mean RT Suicide –mean RT Neutral), resulting in a suicide-specific interference score. Similarly, interference scores were calculated for the positive and negative words by subtracting the mean RT of neutral words from the mean RT of positive and negative words.

**Results:**

A Group × Interference ANOVA showed a significant interaction effect (p <.001, ηp2 = .09), indicating that group effects significantly vary across interference type. *Post hoc* comparisons revealed that both ideators and attempters demonstrated greater interferences only for suicide-related words compared to healthy controls, indicating a SAB in patients, while a difference between ideators and attempters was lacking. The suicide interference score classified with an AUC = 0.73, 95% CI [0.65 – 0.82], p <.001, between controls and patients with STBs. The M-SST demonstrated good internal consistency and convergent validity.

**Discussion:**

The study adds evidence to the assumptions of the Cognitive Model of Suicide, viewing a SAB as a cognitive marker of suicide vulnerability independently of the engagement in suicidal behavior. The results’ clinical implications are discussed in the context of recommended intervention strategies during an acute suicidal state. Future studies with the M-SST should include non-suicidal patient controls to investigate whether a SAB is uniquely related to suicidality.

## 1 Introduction

Despite increased research efforts that aim to improve the detection and prediction of suicidal thoughts and behaviors (STBs), the accuracy of suicide risk prediction has not improved significantly over the last fifty years (1, 2). At present, retrospective self-report questionnaires assessing the prevalence of STBs are a commonly used method (3). However, several studies have shown that the use of risk scales failed to adequately capture the risk of STBs (4). This might be related to findings showing that patients deny current suicidal ideation (SI) due to expected negative consequences (5, 6) or report their own suicide risk state with retrospective bias, as suicide risk can fluctuate rapidly (7, 8). In this context, there has been an increasing interest in the development of performance-based measures in order to assess implicit risk markers that are linked to STBs and might be able to additionally inform about a person’s risk status. Besides the potential enhancement of suicide risk evaluation, the role of implicit processes in suicidal trajectories is not yet fully understood and represents a promising avenue for future research. For example, the Dual-System Model of Suicidality (9) posits that maladaptive implicit processes run automatically and unconsciously, are activated in the context of situational factors (e.g. negative events, suicidal trigger, emotional distress), and prevent adaptive coping with suicidal thoughts and urges. Along this line, a study using Ecological Momentary Assessment (EMA) indicates that higher SI variability, which potentially raises risk for suicidal behavior, was associated with a greater deficit in attentional control (10). More interestingly, a mindfulness-based intervention reduced attentional dyscontrol in high suicide risk Veterans, thus demonstrating that implicit risk markers are modifiable (11). In this context, research has increasingly focused on suicide-specific implicit markers such as a suicide attentional bias, which is theoretically linked to the Cognitive Model of Suicide proposed by Wenzel and Beck (12). The model assumes that individuals with an activated suicide schema struggle to direct their attention away from *suicide-related* information (e.g. suicide-related words) because the confrontation with such stimuli activates a suicide-specific network including cognitive processes (suicidal thoughts) and associated emotions, which increases the likelihood of experiencing SI and potential suicide attempts (12).

The Suicide Stroop Task (SST) represents a performance-based measure, assessing an implicit suicide-specific network, wherein an increased response latency on suicide-related words indicates a suicide attentional bias. Cha et al. (13) developed the first computerized SST that was administered to lifetime suicide attempters and non-attempter psychiatric controls. The SST consists of trials with neutral words (museum, paper, engine), negative words (alone, rejected, stupid), positive words (happy, success, pleasure), and suicide-related words (suicide, dead, funeral), which are randomly presented on a screen in red or blue ink. Participants were instructed to select the color of the words by key response as quickly as possible. Based on the reaction times, a suicide-specific interference score (SIS) was calculated by subtracting the mean reaction time (mean RT) for the neutral words from the mean reaction time (mean RT) for the suicide related words (mean RT_Suicide_ – mean RT_Neutral_). Similarly, interference scores were calculated for the positive and negative words resulting in three interference scores. Cha et al. (13) demonstrated that only suicide-related interference was significantly greater among suicide attempters than in non-attempters using independent *t*-tests. However, the authors did not run an additional *Group* × *Interference* ANOVA to account for the repeated measures design, thus potentially limiting the validity of the results. In recent years, the SST was used in several studies with different samples including inpatients (14, 15), college students (16, 17) and community-based samples (18), providing mixed results. In a recent meta-analysis, Wilson et al. (19) investigated the psychometric properties of the SST by comparing adult and adolescent suicide attempters vs. suicide ideators vs. non-suicidal controls. Non-suicidal controls were either psychiatric patients from psychiatric treatment settings or healthy controls recruited from surrounding communities (community sample). Their results failed to show a significant difference in suicide interference between these three groups and demonstrated poor psychometric properties of the SST. Consistent with these results, a recent study with a sample of youth high-risk patients replicated the insufficient psychometric properties of the SST (20). Consequently, some authors started generally questioning whether a suicide attentional bias indeed exists in individuals with STBs (18). However, the inadequate internal consistency of the SST may explain prior findings because unreliable measurements obscure true individual differences. Given the problematic psychometric properties of the SST and the repeated failure of demonstrating a suicide attentional bias, the development of a psychometrically sound SST is of utmost importance. Therefore, we developed a modified Suicide Stroop Task (M-SST) by applying a block-wise design and increasing the number of category-specific trials as it was recommended by Wilson et al. (19). Additionally, we used a microphone instead of keys for measuring reaction times with the aim of reducing potential cognitive interference due to possible key searching behavior. In a first M-SST validation study with healthy controls and inpatients with STBs, Gold et al. (21) found a suicide attentional bias in patients with STBs when testing interferences separately. Additionally, the interference scores of the M-SST demonstrated improved psychometric properties compared to the SST. However, a repeated measures ANOVA failed to show a significant *Group* × *Interference* interaction, thus limiting the generalizability of the results and warranting further research.

### 1.1 Study aims

Based on the results of Gold et al. (21), the first aim of the current study was to investigate a suicide attentional bias in a more fine-grained sample by distinguishing between different phases of the transition from suicidal thoughts to behavior. Therefore, we compared individuals with a recent suicide attempt (suicide attempters), individuals who recently experienced SI but had never engaged in suicidal behavior (suicide ideators), and healthy controls. Based on the Cognitive Model of Suicide (12), we hypothesized that suicide ideators and attempters should display a suicide attentional bias by showing significantly greater interferences for suicide-related words compared to healthy controls, but not for positive and negative words. A suicide attentional bias in ideators and attempters should also be prevalent when comparing interferences within each subgroup. More specifically, the suicide-specific interference in ideators and attempters should be significantly greater compared to the positive and negative interferences. On the contrary, the interferences in healthy controls should not differ significantly. Despite lacking a profound theoretical foundation, prior SST research has repeatedly hypothesized a difference between ideators and attempters in suicide attentional control (13, 15, 20). However, the Cognitive Model of Suicide outlines that a suicide attentional bias represents a *cognitive* marker, *preceding* suicidal ideation *and* the engagement in suicidal behavior. Accordingly, ideators and attempters should not differ in their degree of a suicide attentional bias. Due to the existing empirical and theoretical inconsistencies, we refrained from formulating a directional hypothesis regarding the difference between ideators and attempters in suicide attentional control.

The second aim of the study was to add evidence to the psychometric properties of the M-SST found by Gold et al. (21). As in the study by Gold et al. (21), we analyzed the utility of stimuli used in the M-SST by including an evaluation of word material, which assessed how strongly aroused participants felt by each word category and how positively versus negatively they evaluated each word. However, compared to Gold et al. (21), who used an extended version of the M-SST with a suicide-related negative word category (e.g. suicide, destruction) and an additional suicide-related positive word category (e.g. suicide, relief), we used the M-SST with only the suicide-related negative word category. The results of Gold et al. (21) had indicated that the suicide-related negative word category (AUC = 0.72) was more selective compared to the suicide-related positive word category (AUC = 0.62) between healthy controls and patients with STBs.

## 2 Materials and methods

### 2.1 Participants and procedure

Participants were recruited into one of three groups: (i) psychiatric inpatients who were hospitalized due to a current suicide attempt (suicide attempters), (ii) psychiatric inpatients who were hospitalized due to recent suicidal ideation without a lifetime history of suicide attempts (suicide ideators) and (iii) control participants without a history of psychopathology and psychotherapy (healthy controls).

Between September 2020 and May 2023, patients at a psychiatric ward of a German hospital were contacted for study participation, if they fulfilled the inclusion criteria of being hospitalized due to a) a current suicide attempt or b) recent suicidal ideation without recent or lifetime suicide attempts and c) being aged ≥ 18. Exclusion criteria included inability to speak or write German fluently, presence of cognitive impairment, color blindness, dyslexia and being currently psychotic. After checking exclusion criteria, n = 40 suicide attempters and n = 40 suicide ideators could be included in the study. After patients agreed to participate, a research assistant provided information about the purpose of the study, the voluntary nature of their participation and data storage. If patients provided informed consent, an appointment for the test session was scheduled and participants received a battery of self-report questionnaires, which they had to complete until the test session. Patients also gave informed consent to access their diagnoses in their medical records. At the scheduled appointment, the test session took place in the research assistant’s office on the psychiatric ward. During the test session, patients completed the M-SST, followed by a questionnaire assessing the evaluation of the presented word stimuli of the M-SST. Finally, a short version of the Suicidal Thoughts and Behaviors Interview (22) was administered.

The control group was recruited via flyers from local communities. Individuals were eligible, if they were a) aged ≥ 18 years and were b) without a history of psychopathology including STBs and psychotherapy. Potential participants of the control group were screened via telephone regarding the exclusion criteria, which included inability to speak or write German fluently, the presence of cognitive impairment, color blindness, dyslexia, a history of psychopathology and psychotherapy including a history of suicidal ideation or suicide attempts. If participants agreed to participate, informed consent and self-report questionnaires were sent by mail and an appointment for the laboratory session was scheduled. After obtaining informed consent, control participants attended the session in the lab of the Department of Medical Psychology and Medical Sociology at the University of Leipzig. The procedure was identical to that for patients except that a short version of a German diagnostic interview for mental disorders [Mini-DIPS, (23)] was administered instead of the SITBI to double check exclusion criteria, which also included screening questions regarding current STBs. Eventually, n = 61 healthy controls could be included in the study. Every participant received 30 € as compensation. All procedures were approved by the ethics committee of the Medical Faculty of the University of Leipzig [012/19-ek].

### 2.2 Measures

#### 2.2.1 Beck Depression Inventory (BDI-II)

The German version of the revised Beck Depression Inventory (24, 25) was used to assess the severity of depression over the previous two weeks. The BDI-II contains 21-items describing depressive symptoms that are to be rated on a 4-point scale (0 to 3). Total scores range from 0 to 63 with higher scores indicating greater depression severity. The internal consistency in our sample was high with Cronbach’s α = .97.

#### 2.2.2 Beck Hopelessness Scale (BHS)

Hopelessness was assessed with the German version of the Beck Hopelessness Scale (26, 27) which comprises 20 true-false items that assess hopeless and pessimistic cognitions. Good reliability and validity have been shown for the BHS (28). Total scores range from 0 to 20 with higher scores indicating stronger hopelessness. The internal consistency in our sample was high with Cronbach’s α = .92.

#### 2.2.3 Beck Scale for Suicide Ideation (BSS)

Suicidal ideation during the previous week was assessed using the German version of the Beck Scale for Suicidal ideation (29, 30). The BSS consists of 21 items and assesses the severity of suicidal symptoms using statement groups on a 3-point scale (0 to 2). Two filter questions (the statement groups four and five) assess the presence of active or passive suicidal thoughts. If participants endorse one of them (i.e., choose a sentence rated 1 or 2), they are to complete the subsequent 14 statement groups which allow for an assessment of the severity of existing suicidal ideation. If participants choose the response option rated “0” for both item 4 and item 5, they skip items 6 to 19 and precede to the last two statement groups. These last two items address frequency and intensity of former suicide attempts and are again to be answered by all participants. They are not part of the total BSS score. It has shown good internal consistency and construct validity (30). Total scores range from 0 to 38 with higher scores indicating greater suicidality. The internal consistency in our sample was high with Cronbach’s α = .89.

#### 2.2.4 Self-Injurious Thoughts and Behaviors Interview (SITBI)

The German version of the Self-Injurous Thoughts and Behaviors Interview (22) is a structured interview and assesses the frequency and intensity of the patients’ suicidal thoughts, plans and behavior. We only administered the sections “suicidal thoughts”, “plans”, and “attempts” to the patients. The SITBI-G has good interrater and retest reliability, as well as good convergent validity (22).

#### 2.2.5 Mini-DIPS

The Mini-DIPS (23) is a short version of a German structured clinical interview for mental disorders. It was used in the control group to verify that the participants had no present or previous mental disorder.

#### 2.2.6 Modified Suicide Stroop Task (M-SST)

For measuring a suicide-specific attentional bias, we administered the Modified Suicide Stroop Task (M-SST) using the E-Prime 3.0 software and the response and stimulus device Chronos (31). The M-SST includes four categories of word stimuli: neutral words and three different categories of emotional words consisting of positive words, negative words, and suicide-related negative words (see **Supplementary Table 1** in the **Supplementary Material** and available at https://osf.io/9ngz3/). To select eligible words for the suicide-related category, we screened public chat histories of suicide online forums of individuals with lived experiences in order to identify words that were negatively associated with STBs. Subsequently, we presented a preselection of words to experts in the field of suicide research and clinicians, who evaluated each word regarding its emotional relevance to patients with SBTs. Based on this evaluation, we conducted a final selection of word stimuli. Each category comprised ten nouns, which were controlled regarding the number of letters and number of syllables. During the M-SST, the ten words of each category were presented in four different font colors (red, yellow, blue, and green) resulting in 40 trials per category, which were presented block-wise. As a result, the M-SST consisted of four experimental blocks with 40 category-specific trials per block. All stimuli were presented on a grey screen of a DELL Latitude Laptop with a screen diameter of 15.6 inches. Participants were instructed to name the font color of the displayed word as quickly and accurately as possible into a microphone, which was connected with the Chronos device. The latter measured the reaction time in milliseconds and provided an audio file with the recorded answer for each trial. Prior to starting the M-SST, a microphone test containing 20 trials (stimuli consisted of words with clothes, e.g., jacket) was conducted in order to test the microphone settings. After the microphone test, the M-SST started with 20 practice trials (words consisted of music instruments) followed by the four experimental blocks. For the experimental blocks of the M-SST, four different block orders were developed, which were randomly distributed across participants in order to avoid position and sequence effects. Each trial started with the presentation of a “+” in the center of the screen for 500 milliseconds (ms) followed by the stimulus, which was displayed on the screen until the microphone registered the participant’s answer. Each trial was limited to a maximum response time of 4000 ms. If no response was registered within this time frame, the reaction time for this trial was automatically set to zero and the trial was excluded. The time between trials was set to 1000 ms. Between each experimental block, participants had a rest of 30 seconds before the next block started automatically. During the administration of the M-SST, the experimenter was blind to the block order and manually registered incorrect responses (naming the wrong font color or reading the word) by using a blind checkbox. Trials with incorrect responses were excluded from the analysis. Outlier response times were defined as response latencies < 200 ms (32, 33) and were excluded before calculating the mean reaction times and interference scores.

#### 2.2.7 Suicide Stroop Survey (SSS)

Following the M-SST, the word material used was evaluated by the participants using the Suicide Stroop Survey (SSS, 21) that was developed by our research team. Participants had to evaluate each word of each category regarding its emotional arousal (“How much did the word affect you emotionally?”) as well as its positive (“How positively do you rate the following words?”) and negative valence (“How negatively do you rate the following words?”). The emotional arousal items were rated on a 10-point Likert scale from *not at all* (1) to *very strong* (10). The same scale was used for the negative and positive valence items from *neutral/not positive* or *neutral*/*not negative* (1) to *very positive* or *very negative* (10). The internal consistency in our sample was excellent with Cronbach’s α = .95 for the arousal scale, and good for the positive valence and negative valence scales with Cronbach’s α = .85.

### 2.3 Statistical analyses

As described in the measures section, trials of the M-SST with incorrect responses, and single outlier response times (< 200 ms, > 4000 ms) defined as invalid trials were excluded from the analyses (21, 32, 33). We decided against the data cleansing procedure used by Cha et al. (13) and prior SST studies (e.g., 18, 19),which removed trials with reaction times (RTs) ± two standard deviations from that participants mean RT, and trials with mean RTs ± two standard deviations from the group mean RT. The procedure has been repeatedly criticized as a critical limitation of prior SST research by increasing the risk of eliminating meaningful data and decreasing the probability of detecting significant effects (18, 19).

#### 2.3.1 Group comparisons in suicide attentional control

For determining group differences, we used interference scores as dependent variables consistent with prior SST research (13, 17, 19, 20). For calculating interference scores, each participant’s raw RTs of the valid trials were averaged, which yielded mean RTs for each word-category specific block (information on means and standard deviations of the mean RTs are included in the **Supplementary Material**, **Supplementary Table 2**). Interference scores were computed by subtracting each participant’s mean RT of neutral words, which served as the reference category, from their mean RT of positive words (Interference_Positive_), negative words (Interference_Negative_), and suicide-related words (Interference_Suicide_).

Group differences in interference scores were calculated in two stages consistent with previous SST research (15, 17, 18). First, we analyzed the interference scores separately by conducting separate *Group* (controls, ideators, attempters) one-way ANOVAs with effect sizes, using each interference score as dependent variable. To additionally account for the repeated measures design, as a second step we performed a repeated measures ANOVA with *Group* (controls, ideators, attempters) as the between-subject factor and *Interference* (positive, negative, suicide) as the within-subject factor. To determine significant group differences, we conducted Bonferroni *post-hoc* pairwise comparisons. To test our hypothesis regarding the within-group pattern of interferences, we calculated repeated measures ANOVAs with *Interference* (Positive, Negative, Suicide) as the within-subject factor for each subgroup. Additionally, we estimated AUC values (area under the curve) using receiver operating characteristics (ROC) analyses to determine the classification accuracy of the interference scores. The study was *a priori* powered to detect medium effect sizes: with a statistical power set at 0.8 and alpha set at 0.05, the one-way ANOVAs could detect an effect size f of >.27 (η_p_^2^ >.07), for the repeated measures ANOVA, f >.22 (η_p_^2^ >.05), which are considered medium effect sizes.

#### 2.3.2 Psychometric properties of the M-SST

For determining the internal consistency of the M-SST, we calculated the split-half reliability (odd- vs. even-numbered trials) with Spearman-Brown correction as in Wilson et al. (19) for the interference scores across the total sample and for each subgroup. For determining the convergent validity of the M-SST, we calculated correlations between the interference scores and with self-report questionnaires using Pearson’s correlation coefficients. Correlation analyses were sufficiently powered (N = 141, α = .05, 1 – β = .95) to detect medium effects (r = .30). For analyzing the evaluation of the stimulus material, we performed separate repeated measures ANOVAs for the arousal, positive and negative valence ratings with *Group* (controls, ideators, attempters) as the between-subject factor and *Word Category* (neutral, positive, negative, suicide) as the within-subject factor. To determine significant group differences, we conducted Bonferroni *post-hoc* pairwise comparisons.

P-values below 0.05 were deemed to be statistically significant. All analyses were performed using SPSS (Version 29.0).

## 3 Results

### 3.1 Descriptive results

Data on sociodemographic and clinical characteristics were missing for one suicide attempter. Sociodemographic and clinical characteristics are presented in **Table 1**. The mean age of the total sample was 31.04 years (*SD* = 12.24) and participants were predominantly female (60.7%). Healthy controls, ideators, and attempters did not significantly differ in age and gender, but in employment, *X^2^* (8, N = 141) = 19.56, *p* = .012, consequently this variable was entered as a covariate in the analyses. Suicide ideators and attempters did not significantly differ in suicidal ideation, depression, and hopelessness. Ideators and attempters differed significantly in their diagnoses *X^2^* (3, N = 79) = 12.59, *p* = .006). Mood disorders were the most common diagnosis in both clinical samples, while personality disorders were more present in attempters, and neurotic-, stress- and somatoform disorders were more present in ideators. Furthermore, ideators indicated a shorter time period of having experienced suicidal thoughts prior to the assessment (*M* = 3.97 days *SD* = 3.87) compared to attempters (*M* = 6.23 days *SD* = 5.40, *t*(69) = 2.03, *p* = .046). Of the patients with a suicide attempt history, *n* = 15 (37.5%) reported a single suicide attempt and *n* = 24 reported two or more suicide attempts (range: 2 – 20). On average, attempters had their index suicide attempt 11.03 days (*SD* = 5.27 days, range 4 – 25 days) prior to the assessment.

**Table 1:** Sociodemographic and clinical characteristics.

	Control group	Suicide ideators	Suicide attempters
Mean (*SD*) age in years	30.54 (13.08)	29.53 (11.13)	33.38 (11.93)
Gender (%)			
Male	37.7	47.6	30.8
Female	60.7	52.4	69.2
Non-binary	1.6	0	0
Employment (%)			
Employed	36.1	27.5	27.5
Unemployed	3.3	25	25
Student	55.7	42.5	42.5
Retired	4.9	5	5
BDI Mean (*SD*)	3.95 (3.83)	37.20 (10.80)	36.62 (11.25)
BHS Mean (*SD*)	2.92 (1.99)	14.85 (4.82)	14.24 (4.55)
BSS Mean (*SD*)	0.03 (0.18)	17.05 (8.99)	18.29 (11.25)
Diagnoses (%)			
Mood disorders	–	55	46.2
Neurotic-, stress- and somatoform disorders	–	35	15.4
Substance use disorders	–	2.5	0
Personality disorders	–	7.5	38.5

BDI, Beck Depression Inventory; BHS, Beck Hopelessness Scale; BSS, Beck Scale for Suicide Ideation. Baseline characteristics except gender are missing for n = 1 suicide attempter.

### 3.2 Data integrity

*N* = 141 M-SST data sets were included, of which 272 trials with an incorrect response (1.21%) and 226 trials (1%) with an outlier response time were removed. Separately analyzed by group, we excluded 227 (2.33%) error trails from a total of 9,760 trials in the control group, 127 (1.98%) error trials from a total of 6,400 trials in the ideators group, and 144 (2.25%) error trials from a total of 6,400 trials in the attempters group. The number of excluded error trials did not significantly differ between groups, *F*(2, 138) = 0.33, *p* = .717.

### 3.3 Group comparisons in suicide attentional control

#### 3.3.1 Between-group analyses

Positive, negative and suicide interference scores of each subgroup are presented in **Table 2**. Analyses of group differences in mean RTs can be found in the **Supplementary Material**, **Supplementary Table 1**. When analyzing interference scores separately, the one-way group ANOVAs with the positive and negative interference scores as dependent variables revealed no significant group differences (see **Table 2**). The one-way group ANOVA with the suicide interference score showed a significant group difference, *F*(2,138) = 9.06, p<.001, η^2^ = .12. Furthermore, the repeated measures ANOVA showed a significant main effect for *Group F*(2, 138) = 5.29, *p* = .006, η_p_^2^ = .07, and for *Interference*, *F*(2, 276) = 31.62, *p* <.001, η_p_^2^ = .19. Moreover, we detected a significant *Group × Interference* interaction, *F*(4, 276) = 6.58, *p* <.001, η_p_^2^ = .09, indicating that group effects significantly vary across interference type. *Post hoc* pairwise comparisons revealed a significant difference for the suicide interference score between controls and suicide ideators (*p* <.001) as well as between controls and attempters (*p* = .004), indicating that controls had a significantly smaller interference for suicide-related words (*M* = 10.23, *SD* = 50.53) compared to suicide ideators (*M* = 120.06, *SD* = 202.79) and attempters (*M* = 105.46, *SD* = 163.71). Ideators and attempters did not significantly differ in their suicide-specific interference (*p* = 1.000). Furthermore, *post hoc* pairwise comparisons for positive and negative interference scores were non-significant (*ps* >.05). We ran the repeated measures ANOVA with employment included as a covariate and the pattern and statistical significance of our effects were unchanged.

**Table 2:** Interference scores across groups.

Score	Control group *n* = 61 *M (SD)*	Suicide ideators *n* = 40 *M (SD)*	Suicide attempters *n* = 40 *M (SD)*	*F*(2,138)	*p*	ES
Interference_Positive_	-4.06 (45.11)	31.70 (101.36)	-1.70 (102.17)	2.59	.079	.04
Interference_Negative_	7.28 (39.69)	50.75 (148.12)	19.79 (142.62)	1.83	.164	.03
Interference_Suicide_	10.23 (50.53)	120.06 (202.79)	105.46 (163.71)	9.06	<.001	.12

M, mean; SD, standard deviation. All M-SST score means and standard deviations are reported in milliseconds (ms).

ES, Effect size (η^2)^.

#### 3.3.2 Within-group analyses

The results of the within-group analyses revealed that the three interference scores of the control group did not significantly differ from each other, *F*(2,120) = 1.95, *p* = .147, η_p_^2^ = .03. In suicide ideators, interference scores differed significantly, *F*(2,78) = 8.95, *p* <.001, η_p_^2^ = .19. Pairwise *post hoc* comparisons between interferences revealed a significant difference between the positive and suicide interference scores (*p* = .004) and the negative and suicide interference scores (*p* = .006), but no significant difference between the positive and negative interference scores ((*p* = .956). This means that suicide ideators displayed a significantly greater interference for suicide-specific words (*M* = 120.06, *SD* = 202.79) compared to interferences for positive words (*M* = 31.70, *SD* = 101.36) and negative words (*M* = 50.75, *SD* = 148.12). In suicide attempters, we could also detect a significant difference between interference scores, *F*(2,78) = 16.64, *p* <.001, η_p_^2^ = .30. Pairwise *post hoc* comparison revealed a significant difference between the positive and suicide interference scores (*p* <.001) and the negative and suicide interference scores (*p* <.001), but no significant difference between the positive and negative interference scores ((*p* = .515). This means that suicide attempters displayed a significantly greater interference for suicide-specific words (*M* = 105.46, *SD* = 163.71) compared to interferences for positive words (*M* = -1.70, *SD* = 102.17) and negative words (*M* = 19.79, *SD* = 142.62).

#### 3.3.3 Classification accuracy

In addition to group comparisons, we conducted classification metrics using area under the curve (AUC) and 95% confidence intervals for interference scores in order to classify between healthy controls and patients with STBs, which were as follows: AUC_Positive_ = 0.57, 95% *CI* [0.47, 0.66], *p* = .18; AUC_Negative_ = 0.55, 95% *CI* [0.45 – 0.64], *p* = .34; and AUC_Suicide_ = 0.73, 95% *CI* [0.65 – 0.82], *p* <.001, indicating that only the suicide-specific interference showed a good classification accuracy in distinguishing patients with STBs from healthy controls. However, suicide-specific interference performed no better than chance in differentiating between ideators and attempters, AUC_suicide_ = 0.51, 95% *CI* [0.39 – 0.64], *p* = .84.

### 3.4 Psychometric properties of the M-SST

#### 3.4.1 Internal consistency

Positive, negative and suicide interference scores demonstrated good to excellent internal consistencies in the total sample and in suicide ideators and attempters (see **Table 3**). In the control group, internal consistency was lower compared to ideators and attempters. Split-half reliabilities of the mean RTs are presented in the **Supplementary Material**, **Supplementary Table 3**.

**Table 3:** Split-half reliability for interference scores across all subjects and subgroups.

Score	All subjects (*N* = 141)	Control group (*n* = 61)	Suicide ideators (*n* = 40)	Suicide attempters (*n* = 40)
Interference_Positive_	.77	.57	.83	.76
Interference_Negative_	.90	.27	.92	.95
Interference_Suicide_	.93	.53	.96	.94

#### 3.4.2 Convergent validity

Inter-correlations between the interference scores and correlations with self-report questionnaires as well as with number of lifetime suicide attempts are presented in **Table 4**. All interference scores were significantly positively associated with each other. Furthermore, the suicide interference score was significantly positively correlated with self-reported depression (BDI), hopelessness (BHS), and suicidal ideation (BSS), while the positive and negative interference scores were not significantly related to self-report clinical measures. Furthermore, the negative interference score revealed a significantly positive association with the arousal score for negative words (SSS_Arou_), indicating that the stronger participants felt emotionally aroused by the negative words used in the M-SST, the greater interference they showed in the block with negative trials. The same was true for the suicide interference score, showing a significantly positive association with the arousal score for suicide-related words (SSS_Arou_), suggesting a greater interference in the block with suicide-related trials when participants felt emotionally aroused by the suicide-related words used in the M-SST.

**Table 4:** Inter-correlations of interference scores and correlations with self-report questionnaires across the total sample (N = 141) and correlations with number of lifetime suicide attempts.

Score	1	2	BDI	BHS	BSS	SSS_Arou_	SSS_Pos_	SSS_Neg_	lifetime SAs^a^
1. Interference_Pos_	1.00	─	.08	.15	.05	.06	.06	–.05	–.07
2. Interference_Neg_	.63^**^	1.00	.15	.17	.13	.22^*^	.00	–.06	–.01
3. Interference_Sui_	.53^**^	.66^**^	.34^**^	.32^**^	.29^**^	.28^**^	.06	–.02	.19

BDI, Beck Depression Inventory; BHS, Beck Hopelessness Scale; BSS, Beck Scale for Suicide Ideation; SSS_Arou_, Rating of the emotional arousal of each word category; SSS_Pos_, Rating of the positive valence of each word category; SSS_Neg_, Rating of the negative valence of each word category; SAs, suicide attempts.

a Correlations were computed for the suicide attempters group only.

^*^p <.01 ^**^p <.001.

#### 3.4.3 Evaluation of the M-SST stimuli

In order to evaluate how strong participants were emotionally aroused by the different word categories, we conducted a repeated measures ANOVA, showing a significant main effect for *Group F*(2, 134) = 3.66, *p* = .03, η_p_^2^ = .05, and for *Word Category*, *F*(3, 402) = 185.12, *p* <.001, η_p_^2^ = .58. The *Group × Word Category* interaction was also significant *F*(6, 402) = 12.58, *p* <.001, η_p_^2^ = .16, indicating that group differences in the arousal ratings differed, depending on the specific word category. *Post-hoc* comparisons revealed a significant difference between controls and ideators for negative words (*p* = .028) and suicide-related words (*p* <.001), indicating that ideators (*M* = 4.65, *SD* = 2.27) were significantly more aroused by negative words than controls (*M* = 3.36, *SD* = 2.26). Ideators (*M* = 6.95, *SD* = 2.32) also displayed a significantly higher emotional arousal for suicide-related words compared to controls (*M* = 3.91, *SD* = 2.74). Attempters differed significantly in their arousal for suicide-related words (*p* <.001) compared to controls by showing a higher arousal for suicide-related words (*M* = 6.39, *SD* = 2.82) than controls (*M* = 3.91, *SD* = 2.74). Ideators and attempters did not significantly differ in their arousal ratings of word categories (*ps* >.05).

For the positive valence ratings, we found a significant main effect for *Word Category F*(3, 399) = 318.82, *p* <.001, η_p_^2^ = .71, but no main effect for *Group F*(2, 133) = 0.50, *p* = .61, η_p_^2^ = .01. There was a significant *Group × Word Category* interaction *F*(6, 399) = 10.19, *p* <.001, η_p_^2^ = .13, indicating that group differences of the positive valence ratings differed, depending on the specific word category. For positive words, *post hoc* comparisons showed a significant difference in the positive evaluation between controls and ideators (*p* <.001) as well as between controls and attempters (*p* <.001). Controls (*M* = 8.69, *SD* = 1.52) evaluated positive words significantly more positively than ideators (*M* = 6.82, *SD* = 2.45) and attempters (*M* = 6.42, *SD* = 2.88). Furthermore, ideators (*p* = .015) and attempters (*p* <.001) significantly differed in their positive valence rating of suicide-related words from controls. Ideators (*M* = 1.66, *SD* = 2.08) and attempters (*M* = 2.21, *SD* = 2.49) evaluated suicide-related words significantly more positively than controls (*M* = 0.63, *SD* = 0.64).

For the negative valence ratings, results showed a significant main effect for *Group F*(2, 133) = 3.57, *p* = .03, η_p_^2^ = .05, and for *Word Category F*(3, 399) = 475.27, *p* <.001, η_p_^2^ = .78. We also detected a significant *Group × Word Category* interaction *F*(6, 399) = 6.70, *p* <.001, η_p_^2^ = .09, indicating that group differences of the negative valence ratings differed, depending on the specific word category. For positive words, *post hoc* analyses revealed that attempters significantly differed from controls (*p* = .016) by showing that attempters (*M* = 1.63, *SD* = 2.26) evaluated positive words significantly more negatively than controls (*M* = 0.52, *SD* = 1.50). For negative words, attempters significantly differed from controls (*p* = .015) by showing that attempters (*M* = 6.09, *SD* = 2.64) evaluated negative words significantly less negatively than controls (*M* = 7.37, *SD* = 1.67). For suicide-related words, attempters significantly differed from controls (*p* <.001) by showing that attempters (*M* = 6.29, *SD* = 2.80) evaluated suicide-related words significantly less negatively than controls (*M* = 8.23, *SD* = 1.94).

## 4 Discussion

### 4.1 Group comparisons in suicidal attentional control

The first aim of the present study was to investigate a suicidal attentional bias in healthy controls, suicide ideators and attempters using a modified version of the SST. Based on the Cognitive Model of Suicide (12), we hypothesized that suicide ideators and attempters would display an attentional bias for suicide-related words in comparison to healthy controls. When analyzing interference scores separately, our results showed a significant group effect for the suicide interference, which was confirmed by an ANOVA accounting for the repeated measures design. Due to the significant interaction effect, groups differed in dependence on interference type. More specifically, ideators and attempters showed greater interferences for suicide-related words compared to controls, which was lacking in positive and negative words. Thus, our hypothesis could be verified in line with the Cognitive Model of Suicide by demonstrating a suicide attentional bias in at-risk individuals with STBs. Our findings of the within-group analyses provide further evidence of a suicide attentional bias in ideators and attempters, showing significantly greater interferences for suicide-related stimuli compared to interferences of positive and negative words that was only found in the two patient subgroups. Contradictory to assumptions of prior SST research, ideators and attempters of the current sample did not differ in their suicide attentional bias. By using the M-SST with improved psychometric properties (21) compared to the prior SST (19, 20), insufficient psychometric properties as a reason for a lack of difference between these subgroups can be excluded. Our results therefore support the assumption of the Cognitive Model of Suicide (12) that a suicide attentional bias serves as an implicit *cognitive* marker of suicidality regardless of whether an individual has ever proceeded from ideation to action. Along this line, the suicide interference score demonstrated an adequate classification accuracy, differentiating between healthy controls and patients with STBs. However, the score was not able to distinguish between suicide ideators and attempters, thus confirming that this score does not differentiate between different stages of the transition from suicidal thoughts to behavior. This is in line with research on other implicit assessment tools, like the Death Implicit Association Test (D-IAT), showing no significant differences between ideators and attempters regarding their implicit associations with death (34, 35).

In terms of risk assessment, a lack of difference in SAB between ideators and attempters found in our study might indicate that the M-SST’s utility in classifying individuals at risk of attempting suicide is limited. However, in the current study the M-SST was administered to inpatients, who stayed in a clinical setting and received medication and treatment. This might have an impact on differences between ideators and attempters. To date, research is lacking on within-person processes and the temporal patterns of a SAB. Therefore, our knowledge is limited as to whether a SAB fluctuates over time and increases in high-risk situations, for example, shortly before a suicide attempt. Instead of focusing solely on between person differences, future studies should further develop behavioral tests to integrate them into an ecological momentary assessment (EMA). This could help to better understand how a SAB and other suicide-specific marker are associated with an individual’s suicide risk and whether they are modifiable through treatment.

### 4.2 The psychometric properties of the M-SST

Our second aim was to assess the psychometric properties of the M-SST in a larger sample compared to the study by Gold et al. (21). Regarding the internal consistency of the M-SST, we found good to excellent reliabilities for the total sample and the patients subgroups, which have been considerably improved compared to results of prior SST studies (19, 20), thus confirming the findings of the validation study by Gold et al. (21). There might be several reasons for this improvement: for example, the blocked stimuli presentation might reduce a possible set-switching bias (18), which represents a response bias occurring when individuals have difficulties switching between different valence categories (e.g., switching from negative to positive stimuli) and might have a potential confounding effect on interference. Moreover, having increased the number of trials might have improved internal consistency. As found by Gold et al. (21), the M-SST demonstrated lower reliabilities for the control group as compared to the patients subgroups. The lower reliabilities are due to the smaller co-variances of interferences in the control group, especially in the block with negative words (see standard deviations in **Table 3**), leading to lower correlations and thus a reduced reliability. The low standard deviations in the control group indicate that the processes of attentional control a more homogeneous in controls compared to ideators and attempters.

In contrast to prior SST studies (17, 18), the suicide interference score of the M-SST demonstrated adequate convergent validity, as it was significantly related to self-reported depression, hopelessness, and suicidal ideation, reflecting a correspondence between explicit clinical measures and an implicit suicide attentional bias. Despite this finding, convergent validity of performance-based measures should not be overestimated by considering potential self-presentation biases of explicit clinical measures and the temporal dynamic of suicide risk (7). Notably, positive, negative and suicide interference scores were significantly inter-correlated. Nevertheless, the content of the suicide-specific category was still specific enough to trigger a suicide attentional bias in patients with STBs.

Besides the assessment of implicit attentional processes, the study included the participants’ evaluation of stimuli used in the M-SST, providing important information regarding the word material’s utility. For the arousal evaluation, results mirrored the interaction effect found in interference scores, revealing that both ideators and attempters were explicitly more aroused by suicide-related words compared to controls, while ideators and attempters did not differ in their arousal ratings. This highlights that the suicide-related words of the M-SST were adequately selected to trigger an implicit suicidal schema by inducing an emotional activation in participants with STBs. Notably, suicide ideators and attempters evaluated the suicide-related words significantly more positively and less negatively compared to controls, demonstrating that individuals with STBs feel attracted to suicide-related content.

### 4.3 Strengths and limitations

The strengths of the present study include its focus on investigating high-risk individuals having experienced recent suicidal thoughts and behavior by dividing them into ideators-only and attempters to differentiate clearly between different stages of the suicidal spectrum. More importantly, we included participants’ evaluation of the word material in order to check its utility and, thus providing valuable information for analyzing and optimizing performance-based tools. However, several limitations of this study should be taken into consideration as well. First, in our sample the time between the present suicidal crisis and M-SST administration was longer compared to prior SST studies (13, 15) whose inpatient samples had been administered the SST within 48 hours after admission. In our study, on the other hand, there were approximately nine days between admission and test administration due to several reasons. For example, all patients had to be tested for COVID-19 after admission, leading to a delay of the initial contact. Furthermore, some patients stated at the initial contact that they needed more time to decide about their study participation. Second, we did not include psychiatric controls without STBs. Thus, we were unable to determine if a suicide attentional bias is uniquely linked to STBs or represents a general marker of psychopathology, which should be addressed in future research. Third, we tested the M-SST in a German sample and participants were rather young in age; the results of the present study may thus not be generalizable to diverse populations and participants of older age. Fourth, our study had a cross-sectional design and no prospective suicide attempt data was available, precluding our ability to determine the predictive validity of suicide-related interference of the M-SST in relation to future suicide attempts. Furthermore, we were not able to examine the temporal dynamics of a suicide-attentional bias and to determine whether a SAB is state-related or behaves trait-like. For example, recent hospitalization for suicide risk as well as treatment of STBs might influence a SAB. Therefore, investigating temporal fluctuations of such an implicit marker is of high clinical relevance and future studies should examine a SAB during hospitalization as well as post-discharge by adapting and integrating the M-SST into EMA to examine critical change processes of this implicit suicidogenic marker. Finally, we used a microphone instead of keys for measuring reaction times with the aim of reducing potential cognitive interference due to possible key searching behavior. This method might be less standardized with regard to the measurement of reaction times and incorrect answers. However, the psychometric properties found by Gold et al. (21) were replicated by using a different sample, which suggests a high test standardization. Nevertheless, future studies should compare the psychometric properties of the M-SST using keys for the potential integration into studies with an online format or an EMA design.

### 4.4 Conclusion and future directions

The study aimed to assess a suicide attentional bias in a suicidal high-risk sample in comparison to healthy individuals who had never experienced STBs by using a modified SST. Our results revealed that the M-SST is able to detect a suicide attentional bias in individuals with STBs and addresses doubts about the existence of a suicide-specific attentional control deficit in individuals with STBs. Our findings support the assumption that a suicide attentional bias can be viewed as an implicit cognitive marker of suicide vulnerability, independently of having exceeded the threshold to suicidal behavior in the past. This finding has noteworthy clinical implications with regard to suicide-risk assessment and commonly recommended strategies for coping with suicidal thoughts and urges.

Given that the incidence of suicidal ideation in the general population is high (36), and many suicide attempts occur among individuals not currently in clinical settings, the M-SST may be a helpful tool for detecting people at risk outside acute clinical settings. Furthermore, the M-SST demonstrated good psychometric properties and, as a next step, future studies with a prospective design should address questions about the predictive validity of the M-SST with regard to future suicide attempts and how the M-SST could be used in combination with other assessments, including self-report measures and clinical expertise.

Besides clinical risk assessment, our results also have clinical implications for the treatment of STBs. As a suicide attentional bias (SAB) indicates, individuals with an activated suicide schema have difficulties to redirect their attention away from suicidal stimuli that may include distressing suicide-related negative affect, suicidal thoughts, and urges. In these situations, different coping strategies tend to be recommended, for example, individuals should distract themselves from their aversive feelings and thoughts through activities or mindfulness, or should focus on something positive in order to cope with suicidal thoughts and urges. However, the question arises, whether, and in which situations, individuals are able to distract themselves from suicidal thoughts and urges, when they exhibit dysfunctional implicit processes, such as an implicit SAB. Individuals with a (high) SAB may not be able to successfully cope with suicidal thoughts and urges due to a biased suicide-specific attention regulation. Therefore, frequently recommended intervention strategies like “self-distraction” or “positive refocusing” should be reconsidered in light of this implicit suicidal mode, as prior studies have shown that distracting from suicidal thoughts and urges was of limited help (37, 38).

To date, our knowledge regarding the influence of implicit processes on suicidal crises in general (e.g. their interaction with emotion-regulation, decision-making and coping with stress during an acute suicidal state), and implicit suicide-specific within-person processes in particular, is limited. Therefore, further research on implicit between- and within processes is urgently needed to gain a more integrative and holistic understanding of a complex problem such as suicidality. By improving the psychometric properties of the Suicide Stroop Task, an important step has been taken in this direction.

## Data availability statement

The raw data supporting the conclusions of this article will be made available by the authors, without undue reservation.

## Ethics statement

The studies involving humans were approved by ethics committee of the Medical Faculty of the University of Leipzig. The studies were conducted in accordance with the local legislation and institutional requirements. The participants provided their written informed consent to participate in this study.

## Author contributions

JB: Writing – original draft, Investigation, Funding acquisition, Formal analysis, Conceptualization. LS: Writing – review & editing. MS: Writing – review & editing, Supervision, Methodology, Conceptualization. HGo: Writing – review & editing, Investigation. TF: Writing – review & editing. KS: Writing – review & editing. HGl: Writing – review & editing, Supervision, Resources.
