# Supplementary material for: A suicide attentional bias as implicit cognitive marker of suicide vulnerability in a high-risk sample
Source: Front Psychiatry. 2024 Aug 7;15:1406675. doi: 10.3389/fpsyt.2024.1406675 (PMC11335530; doi:10.3389/fpsyt.2024.1406675)
Supplement: Supplementary file 1 [file Table_1.docx]

**Table S1**

*Word Material of the M-SST*

| Neutral words | Positive words | Negative words | Suicide-related words |
| --- | --- | --- | --- |
| chair (Stuhl) | security (Sicherheit) | meanness (Gemeinheit) | suicide (Selbstmord) |
| fridge (Kühlschrank) | trust (Vertrauen) | jealousy (Eifersucht) | despair (Verzweiflung) |
| towel (Handtuch) | luck (Glück) | dispute (Streit) | fight (Kampf) |
| shower curtain (Duschvorhang) | hope (Hoffnung) | offense (Beleidigung) | wish to die (Todeswunsch) |
| desk (Schreibtisch) | strengths (Stärke) | laziness (Faulheit) | pain (Schmerz) |
| stove (Herd) | ambition (Ehrgeiz) | difficulty (Schwierigkeit) | destruction (Vernichtung) |
| door handle (Türklinke) | confidence (Zuversicht) | hostility (Feindschaft) | self-hate (Selbsthass) |
| bookshelf (Bücherregal) | friendship (Freundschaft) | damage (Schaden) | end of life (Lebensende) |
| drawer (Schublade) | honesty (Ehrlichkeit) | mistrust (Misstrauen) | leave (Abschied) |
| tap (Wasserhahn) | passion (Leidenschaft) | bad luck (Pech) | failure (Versagen) |
