# Supplementary material for: A suicide attentional bias as implicit cognitive marker of suicide vulnerability in a high-risk sample
Source: Front Psychiatry. 2024 Aug 7;15:1406675. doi: 10.3389/fpsyt.2024.1406675 (PMC11335530; doi:10.3389/fpsyt.2024.1406675)
Supplement: Supplementary file 2 [file Table_2.docx]

**Table S2**

*Mean reaction times across groups*

| Score | Control group  *n* = 61  *M (SD)* | Suicide ideators  *n* = 40  *M (SD)* | Suicide attempters  *n* = 40  *M (SD)* |  |  |  |
| --- | --- | --- | --- | --- | --- | --- |
|  |  |  |  | *F*(2,138) | *p* | ES |
| Mean RT_Neutral_ | 653.02 (111.82) | 758.05 (139.18) | 804.91 (180.91) | 15.31 | < .001 | .18 |
| Mean RT_Positive_ | 648.96 (105.15) | 790.65 (178.77) | 803.21 (197.33) | 15.32 | < .001 | .18 |
| Mean RT_Negative_ | 660.30 (112.92) | 809.69 (207.78) | 824.69 (227.41) | 13.23 | < .001 | .16 |
| Mean RT_Suicide_ | 663.25 (124.86) | 879.00 (269.57) | 910.36 (252.14) | 20.69 | < .001 | .23 |

*Note.* Mean RT = Mean raction time, M = mean, SD = standard deviation. All mean RTs are reported in milliseconds (ms).

ES = Effect size (η^2^ ).
