# Supplementary material for: A suicide attentional bias as implicit cognitive marker of suicide vulnerability in a high-risk sample
Source: Front Psychiatry. 2024 Aug 7;15:1406675. doi: 10.3389/fpsyt.2024.1406675 (PMC11335530; doi:10.3389/fpsyt.2024.1406675)
Supplement: Supplementary file 3 [file Table_3.docx]

**Table S3**

Split-half reliability for scoring approach across all subjects and subgroups

| Score | All subjects  (*N* = 141) | Control group  (*n* = 61) | Suicide ideators  (*n* = 40) | Suicide attempters  (*n* = 40) |
| --- | --- | --- | --- | --- |
| Mean RT_Neutral_ | .97 | .96 | .96 | .98 |
| Mean RT_Positive_ | .98 | .97 | .97 | .97 |
| Mean RT_Negative_ | .98 | .96 | .98 | .98 |
| Mean RT_Suicide_ | .98 | .97 | .99 | .98 |

Note. RT = reaction time.
